# Comparative Safety and Volume Trends in Gastrostomy Tube Placement: Percutaneous Endoscopic Versus Percutaneous Radiologic Approaches at a Single Center

**DOI:** 10.3390/jcm15082812

**Published:** 2026-04-08

**Authors:** Yazan Omari, Bradley Kapten, Saif Affas, Dima Sallam, Serge Sorser, Leonid Shamban

**Affiliations:** 1 Department of Internal Medicine, Henry Ford Providence Hospital, Southfield, MI 48075, USA; 2 Department of Gastroenterology, Henry Ford Providence Hospital, Southfield, MI 48075, USA

**Keywords:** percutaneous endoscopic gastrostomy, percutaneous radiologic gastrostomy, gastroenterology, interventional radiology, complications, length of stay, volume trends

## Abstract

**Background:** Percutaneous endoscopic gastrostomy (PEG) and percutaneous radiologic gastrostomy (PRG) are established techniques for long-term enteral access. Contemporary comparisons of complication patterns, length of stay (LOS), and utilization trends remain limited. **Methods:** We conducted a retrospective cohort study of adult patients undergoing initial gastrostomy placement at a single academic center between 2021 and 2024 (*n* = 341). The primary outcome was any 30-day procedure-related complication. Secondary outcomes included complication subtypes, LOS, and procedural volume trends. Multivariable regression analyses were performed to adjust for potential confounders. **Results:** Among 341 patients, 195 underwent PEG and 146 PRG. Overall complication rates were similar (PEG 16.4% vs. PRG 14.4%, *p* = 0.31). Infectious complications were numerically higher with PEG (4.1% vs. 1.4%), though not statistically significant. Mean LOS was 3.2 days for PEG and 2.8 days for PRG (*p* = 0.12). On multivariable analysis, gastrostomy technique was not associated with complications (aOR 0.88, 95% CI 0.48–1.61) or LOS. PRG utilization increased substantially over the study period, comprising 60.7% of procedures by 2024. **Conclusions:** PEG and PRG demonstrated no statistically significant differences in safety outcomes, with no statistically significant differences in complications or LOS. A marked shift toward PRG utilization was observed over time. These findings support individualized, patient-centered selection of gastrostomy technique, while acknowledging limited power to detect small but clinically meaningful differences.

## 1. Introduction

Gastrostomy tube placement for long-term enteral nutrition is a fundamental procedure in patients with functional gastrointestinal tracts but inadequate oral intake, serving as a cornerstone of supportive care for a diverse patient population [1,2]. Since its introduction in 1980 by Gauderer and Ponsky, percutaneous endoscopic gastrostomy (PEG) has been the cornerstone technique, traditionally performed by gastroenterologists at the bedside or in the endoscopy suite [3]. Over subsequent decades, interventional radiologists developed percutaneous radiologic gastrostomy (PRG) as an alternative approach, utilizing fluoroscopic guidance without the need for endoscopy [4,5]. This method offers a valuable option for patients with obstructing head and neck cancers, esophageal strictures, or other anatomic challenges that preclude safe endoscopy.

The comparative safety profile of PEG versus PRG remains an important clinical question for referring physicians, patients, and healthcare systems. While meta-analyses and large population-based studies suggest similar major complication rates, granular data on specific complications—such as site infection, tube dysfunction, or procedural success—are less consistently reported and can vary significantly between institutions [6,7,8,9]. Furthermore, patient-centered outcomes like length of hospital stay (LOS) post-procedure are critical for healthcare efficiency and patient throughput but are infrequently compared directly between these two techniques in contemporary practice [10].

The COVID-19 pandemic precipitated significant and unprecedented disruptions in procedural services globally. Endoscopy volumes were acutely reduced due to infection control concerns, aerosol-generating procedure precautions, and resource reallocation to pandemic response efforts [11,12]. This period may have accelerated pre-existing trends in procedural redistribution between specialties or created new pathways for patient referral. How this global event has durably influenced the volume, outcomes, and interdisciplinary dynamics of gastrostomy tube placement is not well characterized in the current literature.

This study aims to provide a contemporary, single-center analysis comparing PEG and PRG tube placements over a recent four-year period (2021–2024). We evaluated: (1) overall and specific 30-day complication rates, (2) post-procedural length of stay, and (3) temporal trends in procedural volume, with particular attention to changes following the initial acute phase of the COVID-19 pandemic. Our goal is to provide actionable data for clinicians navigating technique selection and for institutions planning enteral access services.

## 2. Materials and Methods

### 2.1. Study Design and Setting

A retrospective cohort study was conducted at Henry Ford Providence Hospital, a 400-bed academic medical center. The study period spanned from 1 January 2021 to 31 December 2024. The study was approved by the hospital’s Institutional Review Board (IRB #HFPH-2024-087) with a waiver of informed consent due to its retrospective nature.

### 2.2. Patient Selection

All patients aged 18 years or older who underwent gastrostomy tube placement during the study period were identified using Current Procedural Terminology (CPT) codes: 43246 for PEG (endoscopic) and 49440 for PRG (radiologic). Only initial gastrostomy placements were included; patients undergoing tube exchanges, replacements, or conversions from another type of enteral access were excluded to ensure a homogeneous cohort for outcome analysis.

### 2.3. Procedural Techniques

PEG: Performed by attending gastroenterologists or gastroenterology fellows under direct supervision, using standard endoscopic technique. Procedures were conducted with the patient under conscious sedation or monitored anesthesia care. The “pull” or “push” method was employed per endoscopist preference, and antibiotic prophylaxis was administered per institutional and societal guidelines [13].

PRG: Performed by attending interventional radiologists or fellows under direct supervision, utilizing fluoroscopic guidance. Patients received conscious sedation and local anesthesia. Gastric insufflation was achieved via a nasogastric tube placed by the radiology team. This was followed by percutaneous puncture of the stomach and tube placement using T-fastener or similar gastropexy techniques when indicated by the operator.

Procedures were performed by multiple attending physicians and supervised trainees across both specialties. No formal operator-level standardization was applied, reflecting real-world clinical practice.

### 2.4. Data Collection

Data were abstracted from the electronic health record (EHR) by two trained reviewers using a standardized case report form and a detailed codebook to ensure consistency. Collected variables included demographics, primary indication for placement, inpatient/outpatient status, procedural details, all documented complications within 30 days, LOS, and year of procedure. Discrepancies in data abstraction were resolved by consensus or by a third senior reviewer.

### 2.5. Procedure Selection and Institutional Practice

The choice between PEG and PRG was not randomized and was based on multidisciplinary clinical decision-making, incorporating patient-specific factors such as anatomical considerations (e.g., obstructing lesions), comorbidities, and procedural availability.

Missing data were minimal (<5% for all variables) and were handled using complete-case analysis.

### 2.6. Post-Procedural Care

Institutional protocols generally supported initiation of enteral feeding within 12–24 h following placement; however, timing was individualized based on clinical status and operator discretion, which may have influenced LOS.

### 2.7. Outcome Definitions

The primary endpoint was the occurrence of any complication within 30 days of the procedure.

Complications were defined a priori as follows:

Infectious: Peristomal erythema or purulence requiring antibiotics, cellulitis, necrotizing fasciitis, or intra-abdominal infection/abscess.

Mechanical: Tube occlusion, leakage around the tube, or inadvertent dislodgement requiring intervention (e.g., replacement, repositioning).

Bleeding: A decrease in hemoglobin of ≥2 g/dL attributable to the procedure, or the need for blood transfusion, surgical, or angiographic intervention.

Visceral injury: Gastric or intestinal perforation, or significant pneumoperitoneum requiring intervention.

Other: Severe procedure-related pain prolonging hospitalization, post-procedure ileus, or any other event directly related to the procedure that prolonged hospitalization or required additional intervention.

Major complications were defined as those requiring invasive intervention (surgery, interventional radiology), admission to the intensive care unit (ICU), or resulting in death. LOS was defined as the number of days from the procedure to hospital discharge.

### 2.8. Statistical Analysis

Descriptive statistics were reported as frequencies and percentages for categorical variables and as means with standard deviations (SD) for continuous variables. The primary comparison was between PEG and PRG techniques. Categorical outcomes were compared using Fisher’s exact test or the Chi-square test with Yates’ continuity correction, as appropriate. The independent samples *t*-test was used to compare mean LOS. Annual comparisons of complication rates between techniques were performed using Fisher’s exact test for years with smaller sample sizes and the Chi-square test for later years. A two-sided *p*-value of <0.05 was considered statistically significant. All analyses were performed using R statistical software (Version 4.2.1, R Foundation for Statistical Computing, Vienna, Austria). To account for potential confounding, multivariable analyses were performed. A logistic regression model was constructed to evaluate the association between gastrostomy technique (PEG vs. PRG) and 30-day complications, adjusting for clinically relevant covariates including age, sex, inpatient status, and primary indication (neurologic dysphagia vs. malignancy vs. other). Adjusted odds ratios (aORs) with 95% confidence intervals (CIs) were reported. For length of stay (LOS), distribution was assessed using the Shapiro–Wilk test. Given mild non-normality, both parametric (independent *t*-test) and non-parametric (Wilcoxon rank-sum test) analyses were performed, with greater emphasis placed on non-parametric results. Additionally, a multivariable linear regression model was constructed to evaluate the independent association between procedure type and LOS, adjusting for the same covariates. A two-sided *p*-value < 0.05 was considered statistically significant.

## 3. Results

### 3.1. Cohort Characteristics

**Figure 1:**
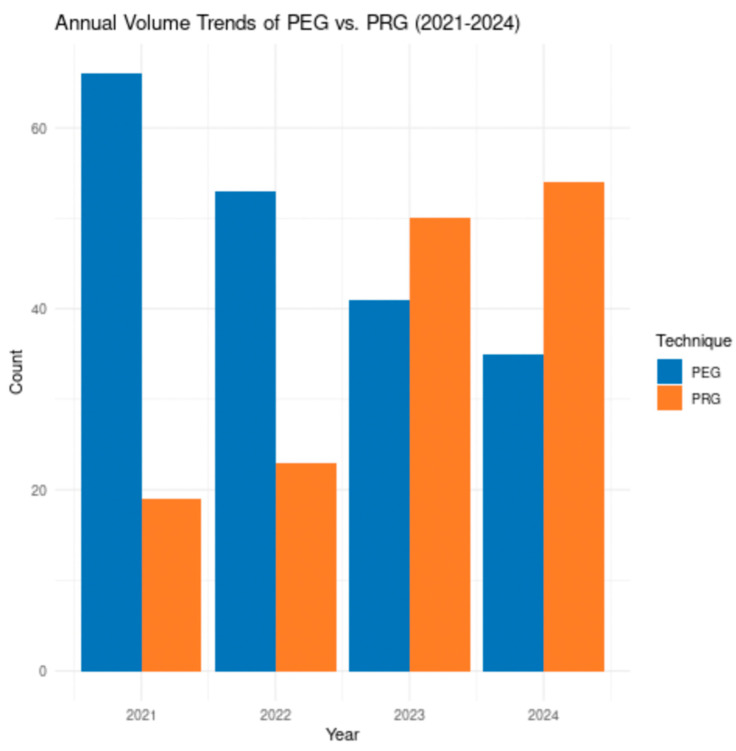
Annual volume trends of PEG vs. PRG placements (2021–2024). Bar chart showing the number of procedures performed each year, stratified by technique (PEG in blue, PRG in orange). Demonstrates the progressive shift from PEG to PRG dominance over the study period.

A total of 341 patients underwent gastrostomy tube placement during the study period (Table 1). The mean age was 68.4 years (SD 12.1), and 184 (54.0%) were male. The majority of procedures were performed on inpatients (92.7%). The most common primary indications were neurologic dysphagia (62.2%) and head/neck malignancy (23.8%). Patient demographics and indications did not differ significantly between the PEG and PRG groups, as shown in Figure 1.

### 3.2. Procedural Volume and Trends

**Figure 2:**
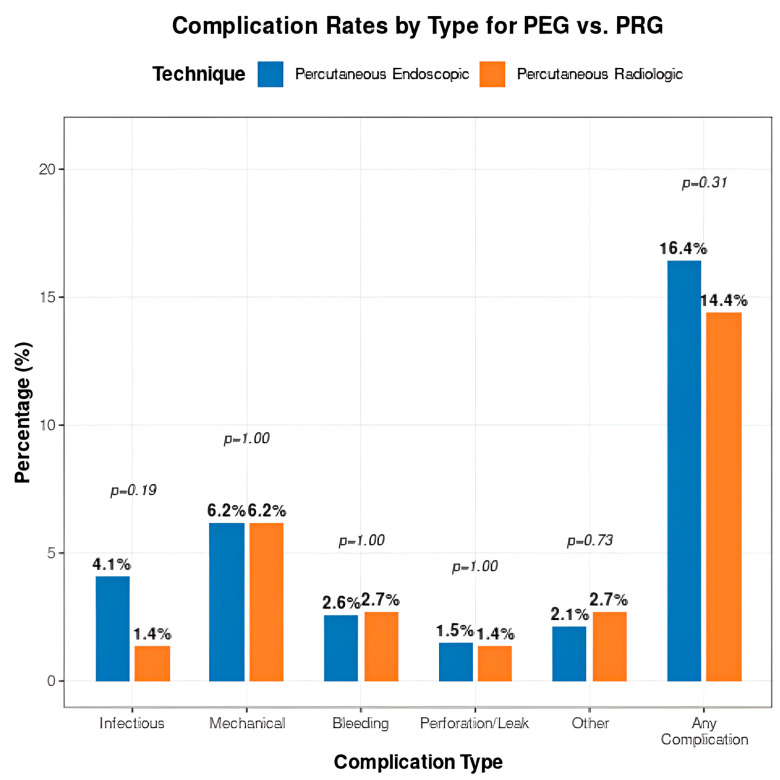
Complication rates by type for PEG vs. PRG. Grouped bar chart comparing rates of infectious, mechanical, bleeding, perforation/leak, and other complications between PEG (blue) and PRG (orange) techniques.

Of the 341 procedures, 195 (57.2%) were PEG and 146 (42.8%) were PRG. A pronounced shift in volume distribution occurred over time (Table 2). PEG volume decreased progressively from 66 cases in 2021 to 35 cases in 2024 (a 47% reduction). Conversely, PRG volume increased from 19 cases in 2021 to 54 cases in 2024 (a 184% increase). By 2023, PRG surpassed PEG in annual procedure volume, comprising 54.9% of gastrostomy tubes placed that year, a trend that continued in 2024 (60.7%) as shown in Figure 2 (Table 2).

### 3.3. Overall and Specific Complication Rates

The overall complication rate within 30 days was 15.5% (53/341). The rate was 16.4% (32/195) for PEG and 14.4% (21/146) for PRG; this difference was not statistically significant (*p* = 0.31). The distribution of specific complication types is detailed in Table 3. Infectious complications, predominantly superficial site infections, occurred in eight PEG patients (4.1%) and two PRG patients (1.4%), a difference that approached but did not reach statistical significance (*p* = 0.19). Rates of mechanical complications, bleeding, and other adverse events were similar between groups. The low bleeding rates observed in both groups (2.6% for PEG, 2.7% for PRG) are consistent with prior reports suggesting that bleeding risk may be more strongly associated with medication use than procedural technique [14].

### 3.4. Length of Stay

**Figure 3:**
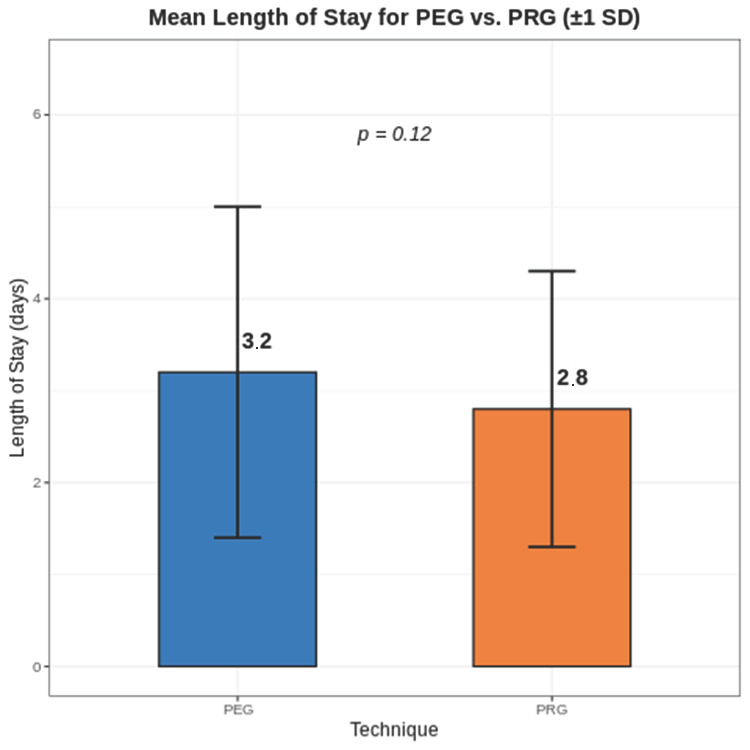
Length of stay distribution for PEG vs. PRG. Box plot showing the distribution of post-procedure length of stay (days) for PEG (**left**) and PRG (**right**) groups, with median, interquartile range, and individual data points.

The mean post-procedure LOS for the entire cohort was 3.0 days (SD 1.7). For patients undergoing PEG, the mean LOS was 3.2 days (SD 1.8), compared to 2.8 days (SD 1.5) for PRG. This 0.4-day difference trended toward significance (*p* = 0.12) as shown in Figure 3 (Table 4).

Results were consistent when analyzed using the Wilcoxon rank-sum test (*p* = 0.15).

The mean difference in LOS was 0.4 days (95% CI −0.1 to 0.9).

### 3.5. Annual Comparison of Complication Rates by Technique

When analyzed by individual year, there were no statistically significant differences in complication rates between PEG and PRG for the years 2021, 2022, or 2023 (*p* = 1.00 for each). In 2024, the complication rate was 22.9% (8/35) for PEG and 11.1% (6/54) for PRG; this difference was not statistically significant (*p* = 0.23).

### 3.6. Major vs. Minor Complications

Of the 53 total complications, 11 (20.8%) were classified as major and 42 (79.2%) as minor. Major complications included events requiring invasive intervention, ICU admission, or resulting in death. The distribution of major complications was similar between groups (PEG 6.2% vs. PRG 5.5%, *p* = 0.81). Minor complications, including superficial infections and tube dysfunction, comprised the majority of events in both groups.

### 3.7. Multivariable Analysis

After adjustment for age, sex, inpatient status, and indication, gastrostomy technique was not significantly associated with 30-day complications (PRG vs. PEG: aOR 0.88, 95% CI 0.48–1.61, *p* = 0.68). No individual covariate was independently associated with increased complication risk. For LOS, multivariable linear regression demonstrated no statistically significant association between gastrostomy technique and length of stay (β = −0.32 days for PRG vs. PEG, 95% CI −0.78 to 0.14, *p* = 0.17). These findings remained consistent across sensitivity analyses using non-parametric testing.

The absolute difference in overall complication rates between groups was 2.0% (95% CI −5.8% to 9.8%).

## 4. Discussion

This study demonstrates that PEG and PRG are associated with no statistically significant differences in safety outcomes in contemporary clinical practice, with no statistically significant differences detected in overall or major complication rates. These findings are consistent with prior meta-analyses and population-based studies, which have similarly reported broadly equivalent outcomes between techniques [6,7,9,15,16] We observed a numerically higher rate of infectious complications in the PEG group, as shown in Figure 2 (4.1% vs. 1.4%), although this difference did not reach statistical significance. This trend is biologically plausible, as the PEG technique involves transoral passage of the tube, potentially introducing oropharyngeal flora [17]. However, given the limited number of events and lack of statistical significance, this finding should be interpreted cautiously and considered hypothesis-generating [13]. A modest, non-significant trend toward shorter LOS was observed in the PRG group (2.8 vs. 3.2 days) as shown in Figure 3. While this may reflect differences in sedation practices or post-procedural workflows, multivariable analysis did not demonstrate an independent association between technique and LOS. These findings suggest that factors beyond procedural approach likely play a larger role in determining hospitalization duration. A substantial shift in procedural volume toward PRG was observed over the study period. PRG volume increased by 184%, while PEG volume declined by 47%, with PRG accounting for the majority of procedures by 2024. While this temporal trend coincides with the post-pandemic period, our study design does not allow for causal inference. The absence of pre-pandemic data and formal time-series analysis limits attribution to specific system-level factors. As such, this finding should be interpreted as descriptive and hypothesis-generating. Patient selection for gastrostomy placement remains critical, as prior studies have demonstrated variable outcomes based on underlying diagnosis, particularly in patients with dementia [18] Although cost was not directly assessed, differences in LOS and procedural logistics may have implications for healthcare resource utilization. Prior studies have suggested potential cost differences between techniques, though findings remain variable. Future studies incorporating cost analyses are warranted. Our findings are consistent with recent population-based analyses demonstrating similar complication rates between PEG and PRG, further supporting the generalizability of these observations across practice settings [9]. This study has several limitations. First, its retrospective and single-center design limits generalizability. Second, the non-randomized allocation of PEG versus PRG introduces potential selection bias and confounding by indication, which we attempted to mitigate through multivariable adjustment, though residual confounding remains possible. Third, the sample size may limit the ability to detect smaller but clinically meaningful differences, particularly for less frequent complications. Fourth, reliance on electronic health record documentation may result in underreporting of minor complications. Fifth, A formal a priori power calculation was not performed due to the retrospective design; however, the sample size may be insufficient to detect small but clinically meaningful differences between groups. Sixth, based on the observed event rates, the study was likely underpowered to detect small absolute differences (<8–10%) in complication rates between groups. Finally, the study period lacks a true pre-pandemic baseline, limiting interpretation of temporal trends. 

## 5. Conclusions

In conclusion, this study demonstrates that PEG and PRG are associated with comparable short-term safety outcomes, with no statistically significant differences in complication rates or LOS. Observed differences were modest and not statistically significant, and the study may be underpowered to detect smaller effect sizes. The evolving distribution of procedural volume highlights the importance of adaptable, multidisciplinary approaches to enteral access.

## Figures and Tables

**Table 1:** Baseline patient and procedural characteristics by technique.

Characteristic	PEG (*n* = 195)	PRG (*n* = 146)	*p*-Value
Age, mean ± SD (years)	68.7 ± 12.4	68.0 ± 11.8	0.63
Male sex, *n* (%)	107 (54.9)	77 (52.7)	0.69
Inpatient procedure, *n* (%)	182 (93.3)	134 (91.8)	0.58
Neurologic dysphagia, *n* (%)	123 (63.1)	89 (61.0)	0.69
Head/neck malignancy, *n* (%)	44 (22.6)	37 (25.3)	0.56
Other indications, *n* (%)	28 (14.3)	20 (13.7)	0.88

**Table 2:** Annual procedural volume by technique.

Year	Percutaneous Endoscopic Gastrostomy (PEG)	Percutaneous Radiologic Gastrostomy (PRG)	Total
2021	66 (77.6%)	19 (22.4%)	85
2022	53 (69.7%)	23 (30.3%)	76
2023	41 (45.1%)	50 (54.9%)	91
2024	35 (39.3%)	54 (60.7%)	89
Total	195 (57.2%)	146 (42.8%)	341

**Table 3:** Specific complication rates by technique.

Complication Type	PEG (*n* = 195)	PRG (*n* = 146)	*p*-Value
Infectious	8 (4.1%)	2 (1.4%)	0.19
Mechanical	12 (6.2%)	9 (6.2%)	1.00
Bleeding	5 (2.6%)	4 (2.7%)	1.00
Perforation/leak	3 (1.5%)	2 (1.4%)	1.00
Other	4 (2.1%)	4 (2.7%)	0.73
Any complication	32 (16.4%)	21 (14.4%)	0.31

**Table 4:** Length of stay after gastrostomy placement (*p* = 0.12).

Technique	Mean LOS ± SD (Days)	Median LOS (IQR)
PEG	3.2 ± 1.8	3 (2–4)
PRG	2.8 ± 1.5	3 (2–3)

## Data Availability

The data that support the findings of this study are available from the corresponding author upon reasonable request.
